# Circulating promastocytes and atypical mast cells in systemic mastocytosis associated with acute myelogenous leukaemia transformation

**DOI:** 10.1002/jha2.250

**Published:** 2021-06-19

**Authors:** Mohammed O. A. Altohami, Dana Lewis, Andrew S. Duncombe

**Affiliations:** ^1^ University Hospitals of Leicester NHS Foundation Trust Leicester UK; ^2^ University Hospital Southampton NHS Foundation Trust Southampton UK

A 72‐year‐old Caucasian male was initially referred with an incidental finding of a raised white cell count of 147.2 × 10^9^/L, haemoglobin of 87 g/L, platelet count of 329 × 10^9^/L and palpable splenomegaly measuring 18 cm on abdominal ultrasound. Differential WBC showed neutrophils 48.6 × 10^9^/L, monocytes 7.4 × 10^9^/L, basophils 1.5 × 10^9^/L, eosinophils 2.9 × 10^9^/L and blasts 8.8 × 10^9^/L. Bone marrow biopsy at the time of progression demonstrated 100% cellularity with a myeloid:erythroid ratio > 10:1 with evidence of mast cell infiltration, monocytosis and dysplastic changes. Mast cells were noted to be round to spindle in shape and mostly single with some paratrabecular cuffing. CD117 and CD25 expression was demonstrated. Cytogenetic analysis demonstrated a normal male karyotype. A *KIT* D816V mutation with allele frequency of 38% was identified on myeloid gene panel profiling. *BCR‐ABL1*, *FIP1L1‐PDGFRA*, *JAK2* V617F, *CALR*, *MPL* and *JAK2* exon 12 mutations were all negative. Surprisingly, mast cell tryptase was only mildly elevated at 26 mg/L. An initial diagnosis of systemic mastocytosis with associated chronic myelomonocytic leukaemia (SM‐CMML) was made, with subsequent rapid progression to acute myelogenous leukaemia.

Blood film images at the time of progression are shown (Figure 1a–d) with atypical mast cells, leukoerythroblastic changes and dysplasia.

**FIGURE 1:**
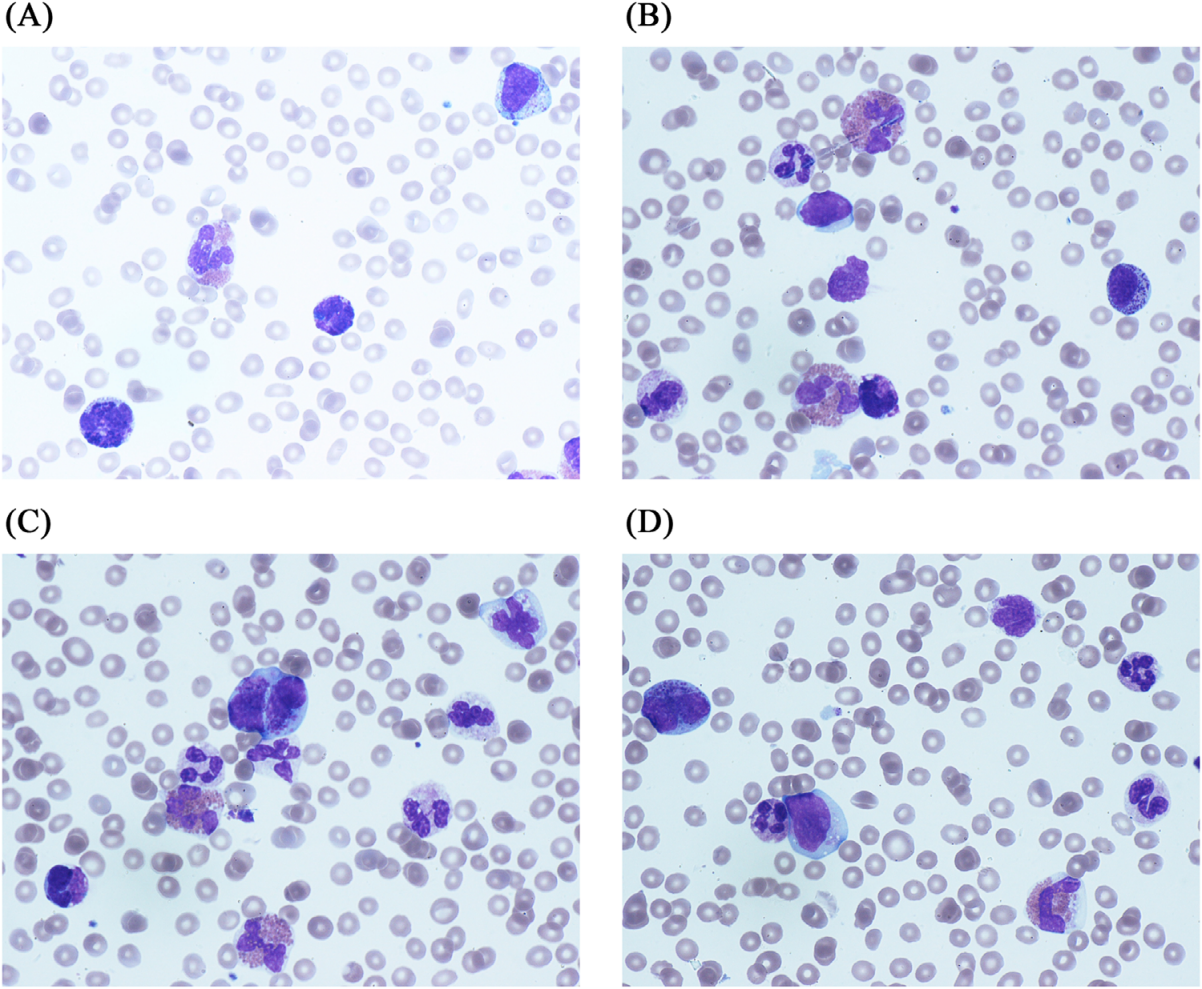
Blood film at the time of progression showing atypical mast cells and dysplastic changes

While normal mast cells are easily identifiable with rounded to oval central nuclei and dense metachromatic granules, which do not obscure the nucleus; atypical mast cells (top right corner of Figure 1a and close to the right border of Figure 1b) can be confused with basophils, monocytes and hairy lymphocytes. Other forms of atypical mast cells include binucleate (also known as promastocytes seen just above the centre of Figure 1c and close to left border of Figure 1d) and hypogranular forms in addition to cells with typical blastic nuclear morphology, which are only identifiable by the presence of metachromatic cytoplasmic granules (centre of Figure 1d). Basophils’ metachromatic granules are larger in size and their nuclei are lobulated (Figure 1a–d).

## CONFLICT OF INTEREST

The authors declare no conflict of interest.

## AUTHOR CONTRIBUTIONS

Mohammed O. A. Altohami captured and wrote the legends of the microscopic images. Dana Lewis and Mohammed O. A. Altohami wrote the first draft of the manuscript. Andrew S. Duncombe was the physician in charge of the patient's care. All authors reviewed and contributed to the final manuscript.

